# Isolation of lignocellulosic biomass-degrading bacteria from *Porcellio dilatatus* gut-enriched cultures

**DOI:** 10.1007/s00253-025-13420-6

**Published:** 2025-02-01

**Authors:** Catarina Coelho, Lígia O. Martins, Igor Tiago

**Affiliations:** 1 Centre for Functional Ecology, Department of Life Sciences, University of Coimbra, 3000-456 Coimbra, Portugal; 2 Instituto de Tecnologia Química e Biológica António Xavier, Universidade Nova de Lisboa, Av. da República, 2780-15 Oeiras, Portugal; 3 Department of Life Sciences, University of Coimbra, 3000-456 Coimbra, Portugal

**Keywords:** *Porcellio dilatatus*, Gut microbiota, LCB-degrading activity-matrix-database, Enriched bacterial cultures, Artificial consortium, Culture-dependent methods

## Abstract

**Abstract:**

The lignocellulosic biomass (LCB) is an attractive, sustainable, and environmentally friendly alternative to fossil sources to produce biofuel, biomaterials, and biochemicals. However, its recalcitrant and heterogenous structure challenges its biodegradation and valorization. The gut microbiome of soil invertebrate species has emerged as a rich source of LCB-degrading bacteria and enzymes in terrestrial ecosystems. The primary objective of this investigation was to identify the bacterial communities within the *Porcellio dilatatus* gut (*Crustacea: Isopods*), to obtain enriched cultures, and to identify bacterial isolates with LCB-degrading activity. A total of 112 enriched cultures were screened, all exhibiting xylanolytic activity. Among them, 94 displayed cellulolytic activity, 30 showed chitinolytic activity, and 21 demonstrated ligninolytic activity. Four enriched cultures were selected, and 128 bacteria with cellulolytic, xylanolytic, chitinolytic, or ligninolytic activity were isolated and taxonomically classified. The obtained results reinforce the potential of bacterial communities within the digestive tract of soil invertebrates as a valuable source of lignocellulose-degrading microorganisms. Thirty-one isolates underwent in-depth enzymatic characterization, and five were selected and functionally evaluated. An artificial bacterial consortium was constructed to assess the potential benefits of using consortia to achieve enhanced LCB degradation. The positive results of this proof-of-concept artificial consortium (PdG-AC) can be used in future applications and is a valuable tool for enzymatic and microbial consortia engineering by, e.g., changing growth conditions for enhanced LCB-degrading abilities.

**Key points:**

*• The gut microbiome of Porcellio dilatatus was characterized.*

*• Porcellio dilatatus gut hosts many lignocellulose-degrading bacteria.*

*• Developed an artificial bacterial consortium for lignocellulose degradation.*

**Supplementary Information:**

The online version contains supplementary material available at 10.1007/s00253-025-13420-6.

## Introduction

The decline of petroleum reserves and the awareness of the non-ecological of these resources for the environment have augmented the need to develop renewable, cost-effective energy and carbon–neutral alternatives (Gírio et al. 2010; Navarro et al. 2018). The biorefinery approach offers a promising solution by converting lignocellulosic biomass (LCB) into value-added products like biochemicals, biomaterials, and biofuels, promoting a sustainable energy future (Rajeswari et al. 2021).

The LCB is a promising renewable resource but poses a challenge due to its complex structure. It is a heterogeneous and recalcitrant matrix composed of carbohydrate polymers of cellulose, hemicellulose, and aromatic lignin polymers (Mohanram et al. 2013). The effective breakdown of this complex material implies using a diverse array of enzymes (Mathews et al. 2015; López-Mondéjar et al. 2016; Cortes-Tolalpa et al. 2017; Janusz et al. 2017). Several natural environments rich in LCB have been explored to find microbes capable of efficiently degrading its components. The gut microbiome of various soil-feeding invertebrate species has emerged as a natural enrichment source of new LCB-degrading enzymes. Diverse bacteria capable of degrading cellulose, hemicellulose, or lignin have been isolated from the digestive tracts of various invertebrate organisms, including wood termites (Suman et al. 2016; Xie et al. 2017; Zhou et al. 2017; Tsegaye et al. 2019), domestic silkmoth *Bombyx mori* (Anand et al. 2010), caterpillars (Gupta et al. 2012), *Holotrichia parallela* larvae (Huang et al. 2012), wood-feeding beetles (Scully et al. 2013), cotton bollworm (Dar et al. 2018), earthworms (Byzov et al. 2009) and beetles (Ceja-Navarro et al. 2019). Kostanjšek et al. (2002) described the terrestrial isopod gut as “hot-spots” of microbial LCB-degrading populations due to their feeding habits and dietary composition. Subsequent scientific works confirmed this potential, with terrestrial isopod species demonstrating promising LCB degradation capabilities (Bredon et al. 2018). In 2022, our research group predicted, by bioinformatic tools, the presence of bacterial genes encoding cellulases, xylanases, and lignin-modifying enzymes within the gut microbiome of *Porcellio dilatatus* giant canyon isopod, a species of woodlouse (Coelho et al. 2022). All those findings imply its potential as a source of LCB-degrading bacteria.

In nature, an effective enzymatic deconstruction of lignocellulosic biomass is performed by the synergistic action of taxonomically distinct microorganisms through the action of multiple enzymes involved in a cascade of LCB-degrading processes (López et al. 2021). However, the in vitro reconstruction of these natural microbial consortia is a key challenge for biotechnology since many LCB-degrading microorganisms remain uncultivable (Taha et al. 2015). Due to the inherent complex structure of LCB, using single-species cultures often exhibits limited LCB-degrading abilities. Therefore, researchers have highlighted the advantages of using bacterial consortia for lignocellulosic degrading processes (Jiménez et al. 2016; Puentes-Téllez and Falcao Salles 2018). Since these microbial communities synergistically optimize intricate regulatory systems, overcoming the problems of feedback regulation and metabolite repression observed in single-species cultures (Wongwilaiwalin et al. 2010; Jiménez et al. 2016). Thus, the premise is that using bacterial consortia will lead to efficient LCB degradation through the division of labor among the selected microbes by producing complementary and more versatile enzymes (Lynd et al. 2002; Deng and Wang 2016). Many artificial bacterial consortia have been successfully developed for other applications such as pyrene degradation (Wanapaisan et al. 2018), biodegradation of crude oil (Srivastava et al. 2022), and polyhydroxyalkanoate (Qin et al. 2022), which emphasizes their biotechnological potential.

This work explored the LCB-degrading potential of the bacterial communities within the *P. dilatatus* gut using dependent-culture methods. Diverse enriched bacterial cultures with degradative activity towards LCB-related substrates were generated. From the most promising enriched cultures, cellulolytic, xylanolytic, chitinolytic, and ligninolytic bacteria were isolated and identified, bearing the potential to be used in engineering artificial LCB-degrading consortium. The LCB-degrading potential of enriched bacterial cultures, isolates and the artificial consortium was evaluated, and results were compiled into enzymatic activity-matrix databases. A proof-of-concept artificial consortium (PdG-AC) was constructed to assess the potential benefits of using consortia to achieve enhanced LCB degradation. An artificial consortium that combines these synergetic capabilities offers a significantly promising approach to improving the rates of LCB degradation (Sadalage et al. 2020). Therefore, an artificial consortium (PdG-AC) was constructed to evaluate the potential benefits of utilizing the synergistic action of different bacteria in lignocellulosic biomass (LCB) degradation. This consortium has proven to be a valuable tool for future enzymatic and bacterial LCB degradation studies.

## Material and methods

### Chemicals and reagents

The enrichment minimal medium (EMM) was composed by 0.1% macronutrients solution concentrated 10x (1 g/L of nitrilotriacetic acid, 0.6 g/L of CaSO_4_.2H_2_O, 1 g/L of MgSO_4_.7H_2_O, 0.8 g/L of NaCl, 1.03 g/L of KNO_3_, 6.89 g/L of NaNO_3_, and 1.11 g/L of NaHPO_4_), 0.01% micronutrients solution concentrated 100x (0.22 g/L of MnSO_4_. H_2_O, 0.05 g/L of ZnSO_4_.7H_2_O, 0.05 g/L of H_3_BO_3_, 0.0025 g/L of CuSO_4_.5H_2_O, 0.0025 g/L of Na_2_MoO_4_.2H_2_O, and 0.0046 g/L of CoCl_2_.6H_2_O) (Tiago et al. 2004) and 0.05% of yeast extract. The solid EMM medium (EMMA) was prepared by adding 15 g/L washed bacteriological agar to the EMM liquid medium. The pH value of the basic composition was buffered for pH 5, 7, and 9 at a final concentration of 50 mM. For pH 5, the acid citric – sodium citrate buffer was used; for pH 7, the Tris–HCl buffer; for pH 9, the sodium carbonate-bicarbonate buffer (Gomori 1955). All buffer solutions were autoclaved separately and were added to the medium after it cooled to 50 °C. To inhibit the fungi growth, 50 mg/L nystatin was added to the minimal enrichment media used for the subculturing process of enriched cultures.

The elemental composition of the diluted Luria–Bertani (L.B.) broth was 5 g/L of tryptone, 2.5 g/L of yeast extract, and 5 g/L g of sodium chloride (NaCl). Carboxymethylcellulose (CMC), xylan from beechwood, colloidal chitin and dry biomass waste mixture, guaiacol, and 2,6-dimethoxyphenol (2,6-DMP) were used as polymeric substrates in the present work. The colloidal chitin was prepared from chitin from shells according to the protocol described by Skujins et al. (1965). The biomass waste mixture composed of wastes of pinewood (50%) and eucalyptus (50%) collected on forests of Oliveira do Hospital County and gently provided by “BLC3- Biomassa Lenho-Celulósica 3ª Geração.” This mixture was dried at room temperature for at least 6 months before grinding and sifting using a hammer mill and a tamper, yielding pieces < 0.42 mm (40 mesh) and > 0.25 mm (40 mesh). In the enzymatic screening on EMMA plates and in the isolation procedure, the biomass waste mixture was substituted by guaiacol and 2,6-DMP.

### Sample collection and dissection

*P. dilatatus* specimens were collected from two cellulosic biomass-rich environments in Coimbra, Portugal: The National Forest of Choupal (40°13′19″N 8°26′46″W) and The Botanical Garden of the University of Coimbra (40°12′22.493″N, 8°25′26.778″W). After collection, to preserve the gut content and numb the host, isopods were stored in plastic boxes and kept on ice until processing at the laboratory. All *P. dilatatus* specimens were anesthetized with a cotton ball soaked in chloroform for 30 min and washed with sterile distilled water to remove any contaminating soil debris (Bolaños et al. 2016). The guts were aseptically extracted firstly by cutting the legs and head with tweezing and a scalpel with the help of a magnifying glass. Later, the guts were separated from the hepatopancreas and cut longitudinally with a scalpel (Drobne et al. 2002). The gut samples were placed into a 1.5 mL microtube containing filtered and autoclaved sterile phosphate-buffered saline (PBS) pH 7.2 and gently vortexed (Vargas-Asensio et al. 2014). The empty gut cell walls were removed from the suspension. The gut content suspensions were cryopreserved by adding glycerol to the suspension at a final concentration of 15%, and tubes were stored at − 80 °C until further use. The guts content from the National Forest of Choupal and the Botanical Garden of the University of Coimbra were used separately as inoculum and were, hereafter, designated as CH and BOT enriched cultures, respectively.

### LCB-bacterial enrichment cultures’ method

Aliquots (200 µL) of *P. dilatatus* gut content 100-fold diluted suspension (with a sterilized PBS solution pH 7.2) were used to inoculate 100-mL Erlenmeyer flasks containing 50 mL of liquid EMM buffered at pH 7 or pH 9, and supplemented with one of the polymeric substrates, namely 0.06% CMC (EMM-CMC), 0.17% xylan from beechwood (EMM-Xyl), 0.50% colloidal chitin (EMM-Chit) or 0.10% biomass waste mixture (EMM-BW). To all previous media, nystatin was added at a final concentration of 50 mg/L to prevent fungi growth. The inoculated Erlenmeyer flasks were incubated in an orbital shaker at 22 °C, 130 rpm for 7 days. A 100-mL Erlenmeyer flask with 50 mL of uninoculated liquid EMM without polymeric substrate was used as a negative control.

After 7 days of incubation, an aliquot (5 mL) of each enriched culture was transferred to fresh liquid EMM supplemented with nystatin, the same substrate, and buffered at the same starting pH value to produce the second generation of enriched cultures. This subculture procedure was repeated seven times more, producing eight generations of enriched bacterial cultures, following the dilution-to-stimulation approach using the pH values and substrates as driving forces (Ho et al. 2012; Diaz-Garcia et al. 2020). The different generations of enriched cultures were identified as Generation 1 (G1) until Generation 8 (G8). The remaining volume of inoculum was used for enzymatic screening in agar plate analyses and to cryopreserve aliquots of the enriched cultures with the addition of glycerol at a final concentration of 15% and storage at − 80 °C till for further use.

### Enzymatic screening of enriched cultures

The cellulolytic, xylanolytic, chitinolytic, and ligninolytic potential of all bacterial enriched cultures (from G2 to G8) was determined based on a semi-quantitative method described by Ventorino et al. (2015) with minor modifications. Briefly, a drop (20 µL) of each bacterial enriched culture with an adjusted optical density of 0.1 at 600 nm (OD_600nm_) was spotted on EMMA plates supplemented with each polymeric substrate at pH 7 and 9. The inoculated EMMA plates were incubated at 25 °C for a maximum of 21 days. Based on preliminary testing results (data not shown), the cellulolytic, xylanolytic, chitinolytic, and ligninolytic enzymes required different incubation length periods for the results to be readable. The cellulolytic and xylanolytic activity of enriched cultures were read after 7 days, while those for chitinolytic and ligninolytic activity were read after 21 days of incubation. The enzymatic potential of the spots was estimated by the index of relative enzymatic activity (IEA), i.e., the difference between the diameter of the clearing zone or halo and the diameter of the spot (in centimeters). The colorimetric tests (described below) were performed in duplicate. The IEA was classified as low (0.10–1.24 cm), medium (1.25–2.50 cm), and high (> 2.50 cm). In the present work, only enriched cultures and isolates with medium and high IEA were considered to have LCB-degrading potential.

Cellulolytic activity in bacterial suspensions was spotted on EMMA plates supplemented with 0.2% CMC (EMMA-CMC) for cellulolytic activity. After the incubation, the agar plates were flooded with 0.1% aqueous solution Congo red for 15 min and then washed with 1 M NaCl for 15 min (Teather and Wood 1982). The cellulolytic enriched cultures were identified by a yellow halo against a red background around the spot (Voget et al. 2006; Gong et al. 2013). Xylanolytic activity in bacterial suspensions was spotted in EMMA plates supplemented with 0.5% xylan from beechwood (EMMA-Xyl). After the incubation, the agar plates were stained with Gram's iodine solution (2.0 g potassium iodide and 1.0 g iodine in 300 mL of distilled water) for 15 min. The presence of a colorless halo represents the xylan hydrolysis (Anand et al. 2010; Meddeb-Mouelhi et al. 2014). For chitinolytic activity, the bacterial suspensions were spotted on EMMA plates supplemented with 1.5% colloidal chitin (EMMA-Chit), which makes the solid medium whiteish mate. The chitin-positive enriched cultures were identified by a clear halo around the spot (Gooday 1990; Suginta et al. 2000). The presence of these halos represents the hydrolysis of β−1,4 glycosidic bonds, present in chitin, in smaller oligosaccharides or monomers (Suginta et al. 2000). The ligninolytic potential of enriched cultures was estimated using only guaiacol as substrate, while the ligninolytic potential of the bacterial isolates was assessed using guaiacol and 2,6-DMP as substrates. The bacterial suspensions were spotted on EMMA plates supplemented with 0.02% guaiacol (EMMA-Gua) or 0.02% 2,6-DMP (EMMA-DMP). The potential ligninolytic activity was evaluated by the appearance of a reddish-brown halo (for guaiacol) or an orange halo (for 2,6-DMP) around the spot as described (Kiiskinen et al. 2004; Lueangjaroenkit et al. 2020; Kimie Okino et al. 2000).

### Isolation of LCB-degrading bacteria

Four enriched cultures were selected for their pronounced enzymatic activity to isolate LCB-degrading bacteria. The selection considered the versatility of the enriched cultures’ enzymatic activity towards the different substrates and the estimated IEA values. The selected enriched cultures were serially diluted, and 100 µL of the last three dilutions (10^−3^–10^−5^) were spread on EMMA plates supplemented with one of the substrates (to which it showed higher enzymatic activity) at different pH values (5; 7; 9) and incubated at various temperatures (20; 30; 40; 50 °C), for 7 days. During incubation, the inoculated agar plates were regularly observed, and all bacterial colonies with different morphologic characteristics were sub-cultured until pure cultures were obtained. All isolates with LCB-degrading activity were cryopreserved in liquid EMM medium supplemented with glycerol 15% and stored at − 80 °C until further use. According to Tanaka et al. (2014), the EMMA was prepared to increase the number of isolates recovered. The various EMMA components were autoclaved separately and mixed after cooling to 50 °C.

### Identification of the isolates by 16S rRNA sequencing and phylogenetic analysis

The molecular identification of the representative bacterial strains was performed by 16S rRNA gene sequencing, followed by its taxonomic assignment. The genomic DNA of each bacterial pure culture was extracted using an in-house adaptation of the (Wiedmann-Al-Ahmad et al. 1994) protocol and used as a template for the PCR assays. Initially, the bacterial isolates were grouped according to their RAPD-PCR (Random Amplified Polymorphic DNA) profile, as previously described by Tiago et al. (2004). The amplification reactions were performed in a thermal cycler (Applied Biosystems, Foster City, CA, USA), using a total volume of 30 µL containing 15 µL MasterMix green (Nzytech, Lisbon, Portugal), 11 µL sterile water, 2 µL of primer OPA-3 10 μM (5′ AGT CAG CCA C 3′) and 2 μL template DNA. The RAPD-PCR protocol included an initial denaturation step (94 °C, 5 min) followed by 35 cycles of denaturation (94 °C, 1 min), annealing (45 °C, 1 min), extension (72 °C, 90 s) steps and a single final extension step (72 °C, 7 min). The RAPD-PCR products were visualized by 2% agarose gel electrophoresis (85 V, 50 min). All bacterial isolates were clustered according to their RAPD pattern profile, and one strain of each RAPD group was selected as a representative strain for further analysis. Each representative strain was assigned with an ID code (PdG1—PdG128) for identification purposes during this study. The representative strains were classified by 16S ribosomal RNA gene phylogenetic analysis. The 16S rRNA gene amplification was carried out using a set of bacterial universal primers 27 F **(**5′ – GAG TTT GAT CCT GGC TCA G – 3′) and 1525 R (5′ – AGA AAG GAG GTG ATC CAG CC – 3′). The amplification reactions were performed using a total volume of 30 µL containing 15 µL MasterMix green (Nzytech, Lisbon, Portugal), 12.5 µL sterile water, 0.25 µL of each primer 50 μM and 2 μL template DNA. The PCR amplification conditions comprised an initial denaturation step (94 °C, 5 min), followed by 30 cycles of denaturation (94 °C, 1 min), annealing (55 °C, 1 min), and extension (72 °C, 2 min) steps, followed by a final extension step (72 °C, 10 min). The 16S rRNA-PCR products were confirmed by 1% agarose gel electrophoresis and purified using a Gelpure Kit (Nzytech, Lisbon, Portugal) according to manufacturer instructions. The purified PCR products were sequenced by Sanger sequencing (Eurofins Genonomics, Ebersberg, Germany) with the primer 519 R, as previously described by Rainey et al. (1996). The 16S rRNA sequence quality was checked using Sequence Scanner software (Applied Biosystems, Foster City, CA, USA), and sequences were edited and assembled using the Bioedit sequence alignment editor (Hall et al. 1999). The partial sequences of the 16S rRNA gene of each representative isolate were used for the basic local alignment search tool (BLAST-n) to determine the taxonomic classification and its closest relatives. This analysis was performed using all 16S ribosomal RNA sequences deposited in the NCBI database. The phylogenetic analysis of the representative strains was carried out using the MEGA (Molecular Evolutionary Genetics Analysis) software version 11. The phylogenetic tree was built using the neighbor-joining algorithm in MEGA software (Tamura et al. 2021).

### Functional characterization: enzymatic profiling, temperature, pH tolerance, and versatility of bacterial strains

The representative strains' cellulolytic, xylanolytic, chitinolytic, and ligninolytic potential was screened following the semi-quantitative agar methods described above. A bacterial suspension in the minimal medium at pH 7 (OD_600_nm adjusted to 0.1) was prepared for each isolate. Then, a drop of each suspension was spotted on EMMA plates supplemented with CMC, xylan, colloidal chitin, guaiacol, or 2,6-DMP. Their enzymatic potential was evaluated across a broad range of incubation temperatures (20, 30, 40 °C) and pH values (5, 7, 9) for the five substrates tested in this work, to determine the most representative strains’ thermal and pH tolerance. The cellulolytic and xylanolytic potential were estimated after 7 days of incubation. The chitinolytic potential was calculated after 21 days of incubation. The ligninolytic potential was assessed after 2 days for EMMA-DMP plates and 21 days for EMMA-Gua plates. The colorimetric tests were performed in duplicate. The results obtained were compiled on an enzymatic activity-based matrix.

### Analysis of LCB-degrading potential of the five isolates as an artificial consortium

Based on their thermal and pH tolerance and the broad enzymatic degrading ability of the representative strains, five strains (PdG38, PdG43, PdG84, PdG85, and PdG94) were chosen to construct a proof-of-concept model of an artificial consortium designated by PdG-AC (*Porcellio dilatatus* gut Artificial Consortium). The cellulolytic, xylanolytic, chitinolytic, and ligninolytic activity of the PdG-AC was evaluated against the performance of the single strains that comprise it. A loopful of overnight cultures was used to inoculate 25 mL of diluted L.B. broth supplemented with the same substrate at which each bacterium was initially isolated (i.e., xylan for PdG38 and PdG43, chitin for PdG84 and PdG85, and guaiacol for PdG94). The Erlenmeyer flasks were incubated in an orbital shaker at 30 °C, 100 rpm, for 24 h. The cells were washed twice with liquid EMM at pH 7 to remove residual components from the growth medium. Then, the washed cells were suspended in a fresh medium and adjusted to a specific OD_600nm_ of 0.1. To construct the bacterial consortium, equal volumes of each isolate (1 mL) were mixed, as previously described in other works by Tao et al. (2017) and Wanapaisan et al. (2018). The enzymatic activity of the PdG-AC and the single strains was evaluated following the same methodology described above. The inoculated EMMA plates were incubated at 30 °C, and the results were read after 2 days for EMMA-DMP, 7 days for EMMA-Xyl plates, 7 days for EMMA-CMC, and 21 days for EMMA-Gua and EMMA-Chit plates. All colorimetric tests were performed in duplicate.

## Results

### The LCB-degrading activity of the enriched cultures

**Fig. 1:**
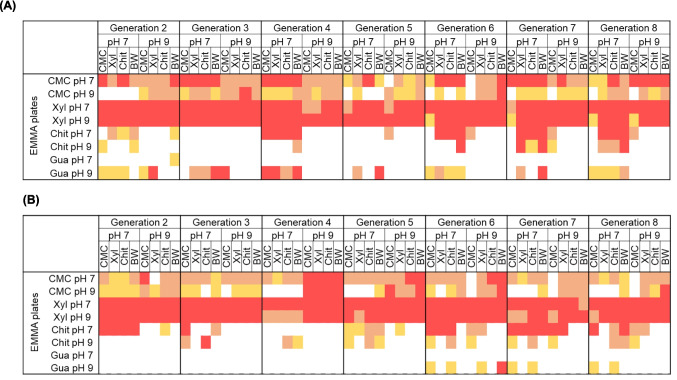
Heatmap of the index of enzymatic activity (IEA) for carboxymethylcellulose, xylan, chitin, and guaiacol of enriched cultures obtained from *P. dilatatus* guts microbiota from **A** National Forest of the Choupal and **B** Botanical Garden of the University of Coimbra. Seven generations (G2-G8) are represented after growing in four substrates (carboxymethylcellulose (CMC), xylan (Xyl), chitin (Chit), and Biomass waste (BW). The IEA was classified as low (halo width 0.10–1.24 cm), medium (halo width 1.25–2.50 cm), and high (halo width > 2.50 cm). The low IEA values are highlighted in yellow; the medium IEA are highlighted in orange, and the high IEA values are highlighted in red

*P.dilatatus* gut content collected from two sites (the National Forest of Choupal and the Botanical Garden of the University of Coimbra, Coimbra, Portugal) was grown on four substrates, CMC, xylan, colloidal chitin, and biomass waste, at pH 7 and 9. Since we aimed at obtaining cultures enriched for degrading different substrates, each culture was transferred to a fresh EMM medium supplemented with the same substrate after 1 week. This way we have reached eight generations and a total of 128 enriched cultures for the target substrates. The first generation (G1) was not considered for analyses, but the remaining generations (G2 to G8), encompassing 112 enriched cultures, were screened to identify LCB-degrading enzymatic activity by spotting these cultures in plates containing carboxymethylcellulose, xylan, chitin, and guaiacol and measuring the halo of degradation after incubation for a period (please see “Material and methods” section). Only those that displayed a medium or a high index of enzymatic activity (IEA), based on the difference between the diameter of the clearing zone or halo and the diameter of the spot (in centimeters) were selected for detailed analysis (Fig. 1). The enriched cultures obtained at pH 7 generally showed higher enzymatic activity for all substrates tested than those obtained at pH 9, except in the case of the degradation of guaiacol. The results indicate that most enriched cultures exhibited high activity regardless of the pH of the enriched culture tested for xylan: all 112 enriched cultures showed in general high enzymatic activity for xylan. Activity for cellulose was also identified in 94 enriched cultures (≈ 83%), but clearly, the enzymatic activity is lower than to xylan, and generally higher activities were measured in cultures grown at pH 7 than in pH 9. Chitinolytic activity was less frequently identified: only 43 enriched cultures (≈38%) showed medium to high IEA values, and most of these (33) were detected at pH 7. These results align with the absence of chitinolytic activity in enriched cultures at pH 9. “Ligninolytic” activity, measured by oxidation of guaiacol, was also mostly detected at pH 9 in cultures supplemented with all of the substrates.

### Isolation and molecular identification of LCB-degrading bacteria

**Fig. 2:**
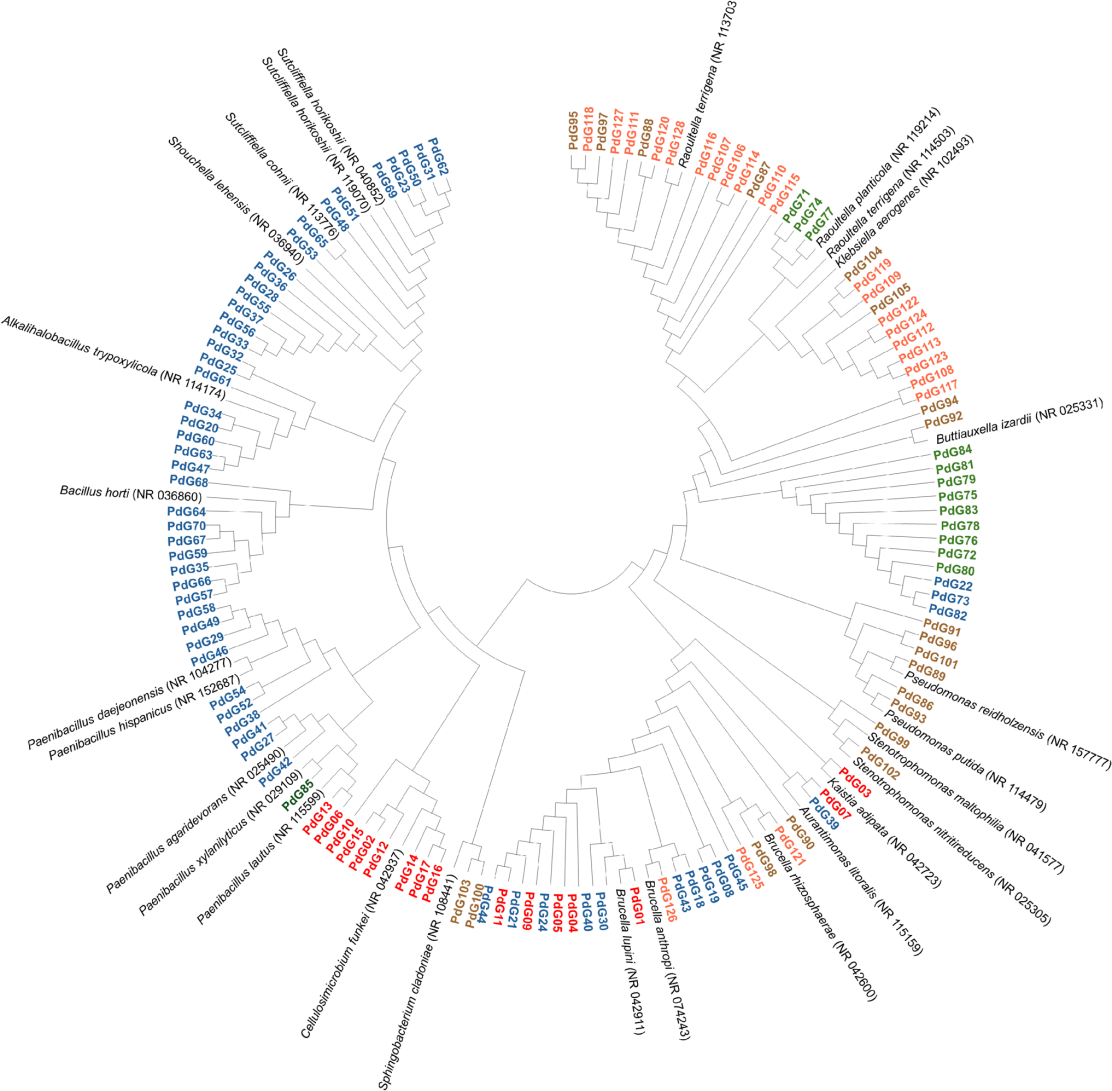
Neighbor-joining phylogenetic tree based on 16S rRNA gene sequences of the 128 representative strains. Isolates recovered for their cellulolytic activity are identified in red, xylanolytic activity in blue, chitinolytic in green, and ligninolytic activity in brown (guaiacol) and orange (2,6-DMP)

Cellulolytic, xylanolytic, chitinolytic, and ligninolytic bacteria were isolated from one of the five enriched cultures exhibiting the highest IEA value for the corresponding substrate (CMC, xylan, chitin, and guaiacol) at each pH value (Supplementary Table S1). These enriched cultures harbored a high diversity of bacteria that could break down cellulose, xylan, chitin, or lignin model compounds. The selection was made considering two factors: the highest activity against multiple substrates and the generation age of the enriched culture (the oldest was preferred over the youngest). This premise allows us to hopefully find promiscuous bacteria with stable genotypes. The phylogenetic distribution of the 128 representative strains and their closest taxonomic relatives are indicated in Fig. 2.

Twenty-six cellulolytic bacteria were isolated from the “CH-XYL-pH 9-G8.” Twelve were isolated from EMMA-CMC plates at pH 9 and fourteen at pH 7, from plates incubated at 30 and 40 °C. Based on RAPD pattern analysis, the 26 isolates were clustered into 17 distinct groups representing 17 strains (PdG01 to PdG17). The taxonomic classification revealed that their closest relatives were members of the *Ochrobactrum*, *Kaistia*, *Paenibacillus, Cellulosimicrobium*, and *Aureimonas* genera*.* One hundred twenty-five xylanolytic bacteria were isolated from the enriched culture “CH-BW-pH 9-G7.” Most isolates (107) were recovered from EMMA-Xyl plates at pH 9, while eighteen were isolated at pH 7, which grew across 20 to 40 °C. The xylanolytic isolates were grouped into 54 representative strains (PdG18-PdG70). The taxonomic classification of these showed that the closest relatives were members of the genera *Bacillus*, *Brucella*, *Paenibacillus*, *Alkalihalobacillus*, *Sutclieffiella, Shouchella*, and *Aureimonas*. Sixteen chitinolytic bacteria were isolated from the enriched culture “CH-XYL-pH 7-G7.” Four isolates were recovered from EMMA-Chit plates at pH 7 and twelve from agar plates at pH 9, that grew at 20 and 40 °C. According to their RAPD analysis, the sixteen chitinolytic isolates were grouped, representing fifteen strains (PdG71-PdG85). The taxonomic classification shows that the closest relatives belonged to the genera *Klebsiella*, *Raoultella*, *Paenibacillus*, and *Aeromonas*. Ligninolytic bacteria were isolated from the enriched culture “CH-BW-pH 7-G5.” Two model lignin-phenolic compounds, guaiacol and 2,6-DMP, were used in the enzymatic screening to enhance the isolation efficiency. Twenty-two isolates were recovered from EMMA-Gua plates, eight from agar plates at pH 5, five at pH 7, and nine at pH 9. Most of the isolates were recovered from agar plates incubated at 20 °C. The RAPD analysis clustered the twenty-two isolates into twenty representative strains (PdG86-PdG105). The taxonomic classification of these strains showed that the closest relatives of these strains belonged to the genera *Brucella*, *Raoultella*, *Ochrobactrum*, *Klebsiella*, *Pseudomonas*, *Buttiauxella*, *Stenotrophomonas*, and *Sphingobacterium.* Thirty-six isolates were recovered from EMMA-DMP plates. Of these, fifteen isolates were isolated from agar plates at pH 5, fourteen isolates at pH 7 and nine at pH 9. Most isolates were recovered from agar plates incubated at 20 and 30 °C. Based on RAPD analysis, the isolates were clustered and represented by twenty-two representative strains (PdG106-PdG128). Taxonomically, the representative strains were closely related to the genera *Ochrobactrum*, *Brucella*, and *Klebsiella* strains*.* The isolates were recovered from EMMA-DMP plates at the three pH values. Still, the enzymatic potential of the isolates for degrading 2,6-DMP was assessed at pH 5 since we observed that 2,6-DMP is more stable under slightly acidic conditions.

### Characterization of LCB-degrading bacteria isolates

**Fig. 3:**
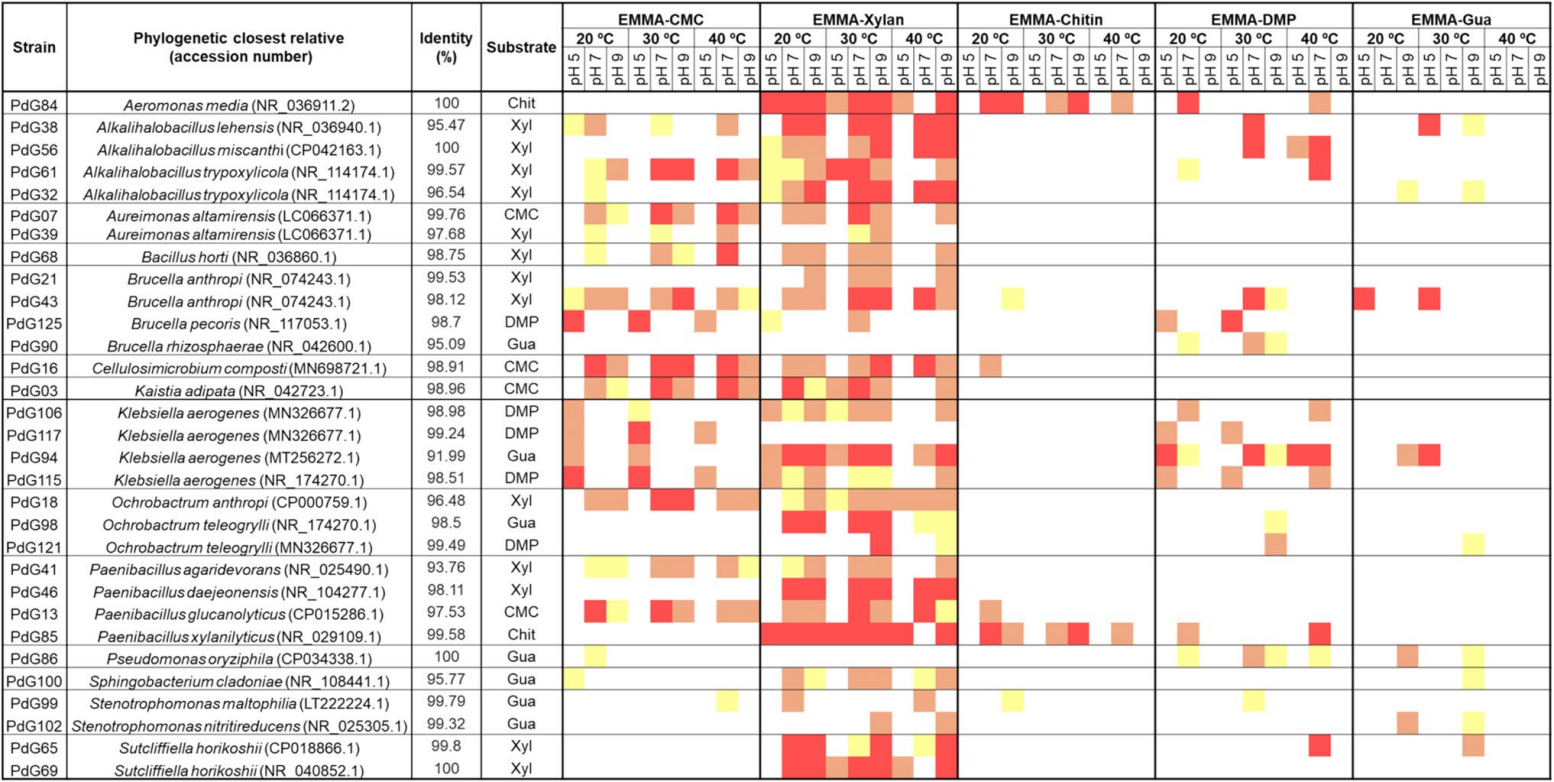
Enzymatic activity matrix of the 31 selected representative strains against five substrates (carboxymethylcellulose (CMC), xylan (Xyl), chitin (Chit), 2,6-Dimethoxyphenol (DMP) and guaiacol (Gua) at three pH values. The IEA (index of enzymatic activity) was classified as low (0.10–1.24 cm), medium (1.25–2.50 cm), and high (> 2.50 cm). The low IEA values are highlighted in yellow; the medium IEA are highlighted in orange, and the high IEA values are highlighted in red

Considering that robustness and multi-enzymatic activity are crucial factors for industrial applications of the LCB-degrading strains, the selection process prioritized strains with the lowest 16S rRNA gene similarity values (Supplementary Table S2) to ensure a good representation of distinct enzymes with LCB-degrading activities. Therefore, strains exhibiting the lowest similarity percentages in their 16S rRNA gene sequence to their closest Blast hit were selected within each genus. Ultimately, 31 out of 128 representative strains were chosen for in-depth enzymatic analysis. The results of this multi-enzyme activity array are summarized in the enzymatic activity matrix presented in Fig. 3. Generally, most strains displayed the highest activity at 30 °C. Regarding pH tolerance, the results revealed that xylanolytic, cellulolytic, and chitinolytic activities were highest at pH 7 and 9 (i.e., neutral to alkaline conditions).

In contrast, the ligninolytic activity was maximal at pH 5 (acidic conditions). These results contradict previous observations regarding LCB-degrading activity observed optimally at alkaline pH 9 (see “The LCB-degrading activity of the enriched cultures” section). However, this has not been investigated here and requires future exploration. Regarding substrate specificity, none of the strains degraded all five substrates tested. However, 22 out of 31 (≈30%) exhibited activity against two or three substrates simultaneously, highlighting their promiscuity in degrading various LCB components.

As expected, xylanolytic activity was the most prevalent among the analyzed strains. Twenty-eight strains (≈ 87% of the selected strains) exhibited a medium to high IEA value for xylan degradation across all temperatures and pH values tested. Interestingly, a significant portion (15 out of 28) of xylanolytic strains also showed cellulolytic activity. The chitinolytic and ligninolytic activities were observed in fewer xylanolytic strains (4 out of 28 and 12 out of 28, respectively). For instance, strain PdG06 showed a higher xylanolytic and cellulolytic activity, highlighting its potential to degrade these specific LCB components. In contrast, strains PdG84 and PdG85 displayed a broader enzymatic profile, exhibiting chitinolytic and ligninolytic activities alongside xylanolytic activity.

The cellulolytic activity was the second most prevalent, observed in 16 of 31 strains (~ 53%). These strains exhibited medium to high IEA values (ranging from 1.25 to 3.75 cm) for degrading CMC across various temperatures and pH values. Interestingly, most cellulolytic strains (9 out of 16) also showed ligninolytic activity. A specific subset of isolates (PdG94, PdG106, PdG115, PdG117) taxonomically closely related to members of the genus *Klebsiella* showed a high cellulolytic activity at pH 5. This unique characteristic suggests that these *Klebsiella*-related strains possess specific enzymatic adaptations, enabling them to function in acidic conditions and potentially providing an advantage in specific ecological niches.

In terms of ligninolytic activity, 15 strains (~ 48%) displayed medium to high IEA values for degrading lignin model compounds (guaiacol and 2,6-DMP). The enzymatic profiles were generally similar across the two lignin model compounds tested, but some variations were observed. Notably, activity against 2,6-DMP was mainly observed under acidic conditions (pH 5), suggesting that these enzymes might be better suited for acidic conditions. Additionally, guaiacol degradation activity exhibited less pH dependence, with strains exhibiting activity across the tested pH range. However, some strains also demonstrated improved performance under acidic conditions. Interestingly, most ligninolytic strains showed enzymatic activity against other substrates. Only two strains with high sequence homology to *Pseudomonas oryziphila* (100%) and *Brucella rhizosphaerae* (95.1%) exhibited exclusively ligninolytic activity.

The chitinolytic activity was the least prevalent among the enzymes analyzed. Only four out of 31 strains (~ 13%) showed medium to high IEA values for chitin degradation; however, all four chitinolytic strains exhibited at least one additional enzymatic activity. For instance, all strains showed xylanolytic activity, and two possessed cellulolytic activity, while the other two displayed ligninolytic activity.

### Selection of strains for the construction of a proof-of-concept artificial LCB-degrading consortium

Following the analysis of the enzymatic activity profiles of the 31 strains (summarized in the matrix-activity database, Fig. 3), several candidates were identified as promising to construct an artificial LCB-degrading consortium as a proof-of-concept: the *Porcellio dilatatus* Gut Artificial Consortium – PdG-AC. The enzymatic versatility criterion encompasses a combination that exhibits a range of enzymatic activities of the individual strains. The two critical criteria that guided the selection of these isolates were their taxonomical affiliation and the enzymatic versatility of the strains. The criteria concerning the taxonomic data encompassed the use of bacteria from different genera to favor a broadened range of enzymatic abilities within the consortium. Furthermore, considering the recalcitrant and heterogenous structure of LCB, the strains exhibiting higher ligninolytic activity were preferentially selected. This prioritization highlights the importance of ligninolytic enzymes for an efficient LCB breakdown. Five strains were selected, namely PdG94 (closest relative to *Klebsiella* genus, 91.99%), PdG84 (*Aeromonas* genus, 100%), PdG85 (*Paenibacillus* genus, 99.58%), PdG43 (*Brucella* genus, 98.12%). Previous studies by Georgiadou et al. (2021) described that one could potentially improve the artificial consortia' ability to degrade various LCB polymers by changing growth conditions and selecting strains from different genera: i.e., these are prone to be engineered accordingly to the feedstocks that need to be degraded/compounds to be produced.

### Evaluation of the enzymatic potential of the artificial consortium PdG-AC

**Fig. 4:**
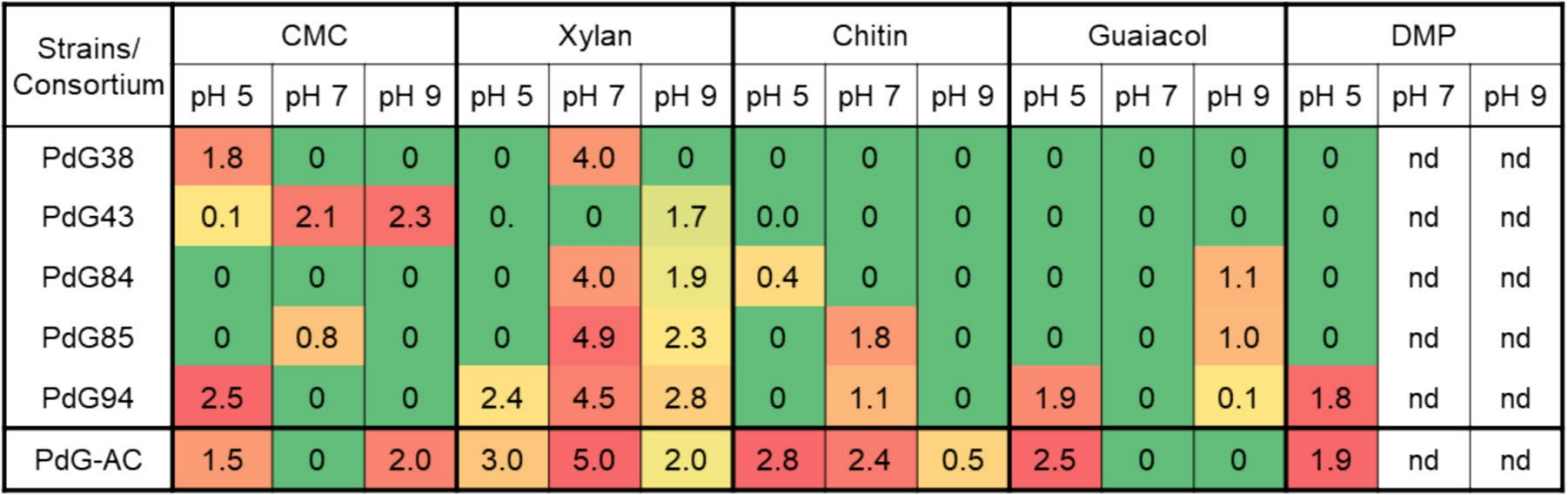
Index of enzymatic activity (IEA) values matrix of the five single strains versus PdG-AC consortium for five substrates, carboxymethylcellulose (CMC), xylan, chitin, guaiacol, and 2,6-DMP at three pH values. The IEA was classified as low (0.10–1.24 cm), medium (1.25–2.50 cm), and high (> 2.50 cm). The low IEA values are highlighted in yellow; the medium IEA are highlighted in orange, and the high IEA values are highlighted in red. nd, not determined

The enzymatic potential of the PdG-AC was assessed using the LCB-degrading activity tests employed previously. In parallel, the LCB-degrading activity of the individual strains was also evaluated, ensuring consistency and allowing direct comparison of the results obtained. Compared to individual strains, the consortium generally showed slightly higher IEA values for cellulolytic, xylanolytic, chitinolytic, and ligninolytic activity (Fig. 4). These findings suggest synergistic interaction between consortium members, leading to enhanced enzymatic activities compared to those observed with the single strains acting independently, and this synergy is very clear for example, in the case of chitin, that is not or barely degraded by the majority of individual strains. Furthermore, unlike some single strains, the PdG-AC exhibited enzymatic activity against all tested substrates achieving comparable or even higher IEA values under similar conditions. Therefore, these results demonstrate that PdG-AC is an excellent proof-of-concept of an artificial consortium prone to be improved for LCB degradation. Culture conditions, such as pH and temperature, based on the targeted LCB substrate, can be easily manipulated in industrial fermentation settings and hold promise for further enhanced enzymatic performance of the PdG-AC consortium.

## Discussion

Developing new strategies for efficient LCB degradation to foster the production of several added-value products is of great industrial interest. Previous works have emphasized the importance of the synergetic and cooperative interactions among microorganisms for an effective LCB breakdown. The main objective of this work was to isolate and characterize LCB-degrading bacteria from *P. dilatatus* gut-enriched cultures with complementary LCB-degrading capacities. A proof-of-concept artificial consortium (PdG-AC) was also constructed to assess the potential advantages of using a consortium to achieve enhanced LCB degradation. The evaluation focused on comparing the enzymatic activity rates of the artificial consortium against the ones of the single strains that composed it.

Among the 112 enriched cultures screened, the highest enzymatic activity was observed in cultures grown at neutral pH, which must relate to the optimal pH of the *P. dilatatus* gut at around pH 7.2 (Zimmer and Topp 1998). For the xylanolytic activity, the high activity across both pH conditions was consistent with the findings of Chakdar et al. (2016), suggesting that the xylanolytic bacteria are highly adaptable to various environments. The enriched cultures with cellulolytic activity from neutral pH appear more versatile than those grown at pH 9, which seem more selective, degrading cellulose only under those more extreme conditions. Chitinolytic and “ligninolytic” activities were observed in fewer enriched cultures. The chitinolytic enriched cultures showed enzymatic activity mostly at pH 7, similarly to guaiacol activity. At the same time, these results highlighted the complexity of isolating ligninolytic bacteria. In future studies, one might need to consider other growth preferences (we have only considered guaiacol as a substrate to test ligninolytic activity breakdown (Falade et al. 2019) when designing ligninolytic screening methods, as Kumar et al. (2020) have already referred. For instance, it could be interesting to explore the use of various dyes structurally comparable to lignin compound models such as Azure B, Phenol red, Remazol Brilliant Blue, and Methylene blue or even lignin in the enriched culture medium to improve the detection of lignin-degrading bacteria (Hooda et al. 2015; Zainith et al. 2019; Singh et al. 2020). Our results indicate that the bacterial communities in *P. dilatatus* gut (at neutral pH) might be better suited for breaking down cellulose, xylan, and chitin (usually performed by hydrolytic enzymes) than lignin (conducted by oxidative enzymes).

Interestingly, many enriched cultures showed considerable enzymatic activity for more than one substrate. For instance, all enriched cultures with cellulolytic activity also exhibited xylanolytic activity, and many of these cultures also exhibited chitinolytic activity. Moreover, diverse xylanolytic and cellulolytic enriched cultures also showed ligninolytic activity, although less frequently (Fig. 1), reinforcing the hypothesis of the potential presence of synergetic and cooperative interactions across the bacterial communities in degrading natural polymeric substrates (Deng and Wang 2016; Cortes-Tolalpa et al. 2017). Several strains were isolated and taxonomically identified from the four most promising enriched cultures. A critical correlation was observed between phylogeny and the enzymatic activity of the 128 representative strains. For instance, the strains isolated with higher ligninolytic potential belonged to families different from those recovered with higher cellulolytic and xylanolytic activity. These observations indicate that different bacterial populations produce distinct sets of enzymes that are especially suited for degrading various components of LCB (Cortes-Tolalpa et al. 2017).

Generally, the taxonomic results obtained in this work align with previous scientific reports (Anand et al. 2010; Brito and David 2009). The identification of cellulolytic, xylanolytic, and chitinolytic strains closely related to *Paenibacillus* strains denotes the enzymatic potential of the members of this genus (Chapla et al. 2012; Rodríguez et al. 2019; Mukherjee et al. 2023). Similarly, the identification of xylanolytic isolates belonging to the genus *Bacillus* and *Alkalihalobacillus* or closely related to the *Shouchella lehensis* strains (previously *Alkalihalobacillus miscanthi* strains) matches prior studies (Zhang et al. 2016; Ma et al. 2020; Masasa et al. 2022). The recovery of chitinolytic and xylanolytic bacteria belonging to the genus *Aeromonas* is consistent with the findings of Anand et al. (2010), who isolated xylanolytic bacteria from *B. mori* digestive tract belonging to the same genus. Finally, the taxonomic classification of the ligninolytic bacteria corroborates the existing knowledge on the activity of genera *Pseudomonas* (Tian et al. 2016; Xu et al. 2022), *Klebsiella* (Bao et al. 2011), *Raoultella* (Hooda et al. 2015), S*tenotrophomonas* (Bao et al. 2011; Ravi et al. 2018; Fathollahi et al. 2021), *Brucella* and *Ochrobactrum* (Taylor et al. 2012; Granja-Travez et al. 2018). Nevertheless, and notably, bacterial strains related to members of genera *Kaistia*, *Aureimonas*, *Brucella*, *Buttiauxella*, and *Stucliffiela*, which are less frequently associated with the degradation of LCB polymers, were also identified in this study. These strains exhibited cellulolytic and xylanolytic activity previously reported (Huang et al. 2012; Chakdar et al. 2016; Xu et al. 2023). Regarding enzymatic versatility, the xylanolytic activity was the most prevalent among the isolates tested, reinforcing the adaptability and versatility of xylanolytic strains reported by Chakdar et al. (2016). Robustness and multi-enzymatic activity are crucial factors for industrial applications of the LCB-degrading strains. The enzymatic versatility and the pH and thermal tolerance of 31 representative strains were assessed. Most of strains displayed the highest activity at 30 °C, a slightly higher temperature than the natural gut temperature of *P. dilatatus,* which is likely closer to 20–22 °C (Yilmaz et al. 2021). Regarding pH tolerance, the xylanolytic, cellulolytic, and chitinolytic activities were highest at pH 7 and 9 (i.e., neutral to alkaline conditions). In contrast, the ligninolytic activity was maximal at pH 5 (acidic conditions). These results contradict previous observations regarding LCB-degrading activity observed optimally at alkaline pH 9 (see “The LCB-degrading activity of the enriched cultures” section). However, this has not been investigated here and requires future exploration.

Several strains showed promising properties for preparing artificial consortium with enhanced LCB-degrading activity. Given this enzymatic versatility five strains from different genera were selected to construct the PdG-AC. This artificial consortium exhibited a clear synergistic interaction among strains, leading to a broader and higher enzymatic activity than those observed with single strains. This is an excellent proof-of-concept of an artificial consortium prone to being engineered for LCB degradation. We envision its utility in industrial fermentation settings, where culture conditions such as pH and temperature can be optimized based on the targeted LCB substrate, potentially enhancing the enzymatic performance of the PdG-AC.

In conclusion, this pioneering effort uses culture-dependent methods to evaluate the *P. dilatatus* gut as a source of LCB-degrading bacteria. The approach employed successive enrichment cultures to facilitate the growth of bacterial populations with cellulolytic, xylanolytic, chitinolytic, and ligninolytic activities. The methodology used to identify promising LCB-degrading bacteria proved effective, and the enriched cultures offer a valuable resource for further exploration of new LCB-degrading bacteria. Several bacterial strains were isolated, exhibiting robust LCB-degrading capabilities, expanding our understanding of the culturable bacterial populations within the *P. dilatatus* gut. An activity-matrix database was established, which proved valuable for successfully selecting promising strains for constructing a functional artificial consortium (PdG-AC), demonstrating greater enzymatic versatility than the individual strains. Future research efforts will focus on the identification and characterization of the specific enzymes responsible for LCB degradation of the newly identified strains and on the assessment of the enzymatic potential of the PdG-AC against natural LCB substrates like wine bagasse, rice straw, wood waste, sawdust or corn stover. These renewable feedstocks provide a more challenging but realistic evaluation of their applicability for LCB degradation and environmental and industrial impact.

## Supplementary Information

Below is the link to the electronic supplementary material.

Supplementary file1 (PDF 477 KB)

## Data Availability

The data that support the findings of this study are not openly available due to reasons of sensitivity and are available from the corresponding author upon reasonable request.
