# Supplementary material for: Isolation of lignocellulosic biomass-degrading bacteria from Porcellio dilatatus gut-enriched cultures
Source: Appl Microbiol Biotechnol. 2025 Feb 1;109:35. doi: 10.1007/s00253-025-13420-6 (PMC11787219; doi:10.1007/s00253-025-13420-6)
Supplement: Supplementary file 1 [file 253_2025_13420_MOESM1_ESM.pdf]

Applied Microbiology and Biotechnology

SUPPLEMENTARY INFORMATION

Isolation of lignocellulosic biomass-degrading bacteria from *Porcellio dilatatus* gut-enriched cultures

Catarina Coelho[1], Lígia O. Martins[2*], Igor Tiago[3*]

[1]*Centre for Functional Ecology, Department of Life Sciences, University of Coimbra, 3000-456 Coimbra, Portugal;*[2] *Instituto de Tecnologia Química e Biológica António Xavier, Universidade Nova de Lisboa, Av. da República, 2780-15 Oeiras, Portugal,* [3]*Department of Life Sciences, University of Coimbra, 3000-456 Coimbra, Portugal*

catarinafc89@gmail.com (C.C.), lmartins@itqb.unl.pt (LOM), itiago@uc.pt (I.T.)

* Corresponding authors

Supplementary Table S1. Top5 of *P. dilatatus* guts enriched cultures with highest activity for each substrate at each pH value. The enriched cultures were constructed using the guts isolated from the isopodscollected in Choupal (CH) and Botanical Garden (BOT). The enzymatic activities were screened against four substrates: carboxymethylcellulose (CMC), xylan , chitin and guaiacol. The activities were measured in enriched cultures different generations (G). Since no cultures showed activity against guaiacol at pH 7, only the top five at pH 9 were considered.

Substrate	Screening condition	Enriched cultures (IEA)
**CMC**	EMMA-CMC pH 7	**CH-Xyl-pH 9-G8 (4.10 cm)**<br>CH-CMC-pH 7-G3 (3.75 cm)<br>CH-Chit-pH 7-G7 (3.75 cm)<br>CH-CMC-pH 7-G2 (3.70 cm)<br>CH-Xyl-pH 7-G3 (3.70 cm)
	EMMA-CMC pH 9	CH-BW-pH 9-G6 (2.85 cm)<br>BOT-CMC-pH 9-G4 (2.85 cm)<br>BOT-BW-pH 9-G8 (2.80 cm)<br>BOT-Chit-pH 9-G4 (2.80 cm)<br>BOT-Xyl-pH 9-G4 (2.75 cm)
**Xylan**	EMMA-Xyl pH 7	CH-CMC-pH 9-G3 (3.85 cm)<br>CH-Xyl-pH 7-G7 (3.85 cm)<br>BOT-CMC-pH 9-G4 (3.85 cm)<br>BOT-Xyl-pH 7-G5 (3.85 cm)<br>BOT-Chit-pH 7-G5 (3.85 cm)
	EMMA-Xyl pH 9	CH-Chit-pH 9-G3 (3.90 cm)<br>CH-Xyl -pH 9-G7 (3.90 cm)<br>**CH-BW-pH 9-G7 (3.90 cm)**<br>BOT-CMC-pH 9-G4 (3.90 cm)<br>CH-CMC-pH 9-G3 (3.85 cm)
**Chitin**	EMMA-Chit pH 7	BOT-Xyl-pH 7-G2 (3.90 cm)<br>BOT-CMC-pH 7-G2 (3.85 cm)<br>CH-CMC-pH 7-G4 (3.80 cm)<br>CH-Xyl-pH 7-G4 (3.80 cm)<br>CH-Xyl-pH 7-G6 (3.80 cm)
	EMMA-Chit pH 9	CH-BW-pH 7-G8 (3.75 cm)<br>CH-BW-pH 7-G6 (3.70 cm)<br>**CH-Xyl-pH 7-G7 (3.70 cm)**<br>CH-BW-pH 7-G7 (3.70 cm)<br>BOT-Chit-pH 7-G3 (2.55 cm)
**Guaiacol**	EMMA-Gua pH 9	**CH-BW-pH 7-G5 (3.55 cm)**<br>BOT-BW-pH 9-G6 (3.40 cm)<br>CH-CMC-pH 7-G4 (3.0 cm)<br>CH-BW-pH 7-G7 (3.0 cm)<br>CH-BW-pH 7-G3 (2.90 cm)

Suplementary Table S2. Summary of the 128 representative strains isolated from enriched cultures with LCB-degrading activity. Isolation conditions and taxonomic affiliation of each strain based on the sequencing of the 16S rRNA gene and Blastn analysis.

Strain	T (ºC)	pH	Description	Query Cover	E value	Per. Ident.(1)	Acc. Len.(2)	Accession
PdG01	20	7	*Brucella lupini* strain LUP21	100%	0.0	99.77%	1477	NR_042911.1
PdG02	30	7	*Cellulosimicrobium funkei* strain W6122	98%	0.0	99.54%	1444	NR_042937.1
PdG03	30	7	*Kaistia adipata* strain Chj404	100%	0.0	98.96%	1413	NR_042723.1
PdG04	30	9	*Brucella lupini* strain LUP21	100%	0.0	99.54%	1477	NR_042911.1
PdG05	30	9	*Brucella anthropi* ATCC 49188	100%	0.0	99.76%	1476	NR_074243.1
PdG06	30	9	*Paenibacillus lautus* strain NRRL NRS-666	99%	0.0	97.54%	1436	NR_115599.1
PdG07	30	9	*Aurantimonas litoralis strain HTCC2156*	96%	0.0	97.39%	1438	NR_115159.1
PdG08	40	7	*Brucella lupini* strain LUP21	100%	0.0	99.31%	1477	NR_042911.1
PdG09	40	7	*Brucella anthropi* ATCC 49188	99%	0.0	99.53%	1476	NR_074243.1
PdG10	40	7	*Cellulosimicrobium funkei* strain W6122	98%	0.0	99.77%	1444	NR_042937.1
PdG11	40	7	*Brucella lupini* strain LUP21	100%	0.0	99.08%	1477	NR_042911.1
PdG12	40	7	*Cellulosimicrobium funkei* strain W6122	95%	0.0	99.77%	1444	NR_042937.1
PdG13	40	9	*Paenibacillus lautus* strain NRRL NRS-666	100%	0.0	97.53%	1436	NR_115599.1
PdG14	40	9	*Cellulosimicrobium funkei* strain W6122	96%	0.0	99.77%	1444	NR_042937.1
PdG15	40	9	*Cellulosimicrobium funkei* strain W6122	96%	0.0	99.77%	1444	NR_042937.1
PdG16	40	9	*Cellulosimicrobium funkei* strain W6122	96%	0.0	99.77%	1444	NR_042937.1
PdG17	40	9	*Cellulosimicrobium funkei* strain W6122	96%	0.0	99.77%	1444	NR_042937.1
PdG18	20	7	*Brucella anthropi* ATCC 49188	87%	0.0	96.89%	1476	NR_074243.1
PdG19	20	7	*Brucella lupini* strain LUP21	100%	0.0	98.85%	1477	NR_042911.1
PdG20	20	9	*Alkalihalobacillus trypoxylicola* strain NBRC 102646	98%	0.0	99.36%	1476	NR_114174.1
PdG21	20	9	*Brucella anthropi* ATCC 49188	100%	0.0	99.53%	1476	NR_074243.1

PdG22	20	9	*Aeromonas media* strain ATCC 33907	99%	0.0	100.00%	1460	NR_119041.1
PdG24	20	9	*Brucella anthropi* ATCC 49188	99%	0.0	99.76%	1476	NR_074243.1
PdG25	20	9	*Alkalihalobacillus trypoxylicola* strain NBRC 102646	98%	0.0	99.57%	1476	NR_114174.1
PdG26	20	9	*Shouchella lehensis* strain MLB2	100%	0.0	98.96%	1525	NR_036940.1
PdG27	20	9	*Paenibacillus agaridevorans* strain DSM 1355	100%	0.0	93.78%	1509	NR_025490.1
PdG28	20	9	*Shouchella lehensis* strain MLB2	100%	0.0	99.35%	1525	NR_036940.1
PdG29	20	9	*Paenibacillus hispanicus* strain AMTAE16	100%	0.0	97.78%	1473	NR_152687.1
PdG30	30	7	*Brucella anthropi* ATCC 49188	100%	0.0	100.00%	1476	NR_074243.1
PdG31	30	9	*Sutcliffiella horikoshii* strain DSM 8719	99%	0.0	99.39%	1521	NR_040852.1
PdG32	30	9	*Alkalihalobacillus trypoxylicola* strain NBRC 102646	96%	0.0	96.54%	1476	NR_114174.1
PdG33	30	9	*Shouchella lehensis* strain MLB2	99%	0.0	99.17%	1525	NR_036940.1
PdG34	30	9	*Alkalihalobacillus trypoxylicola* strain NBRC 102646	98%	0.0	99.78%	1476	NR_114174.1
PdG35	30	9	*Bacillus horti* strain K13	98%	0.0	99.16%	1512	NR_036860.1
PdG36	30	9	*Shouchella lehensis* strain MLB2	99%	0.0	99.15%	1525	NR_036940.1
PdG37	30	9	*Shouchella lehensis* strain MLB2	99%	0.0	99.36%	1525	NR_036940.1
PdG38	30	9	*Shouchella lehensis* strain MLB2	100%	3e-117	95.47%	1525	NR_036940.1
PdG39	30	9	*Aurantimonas litoralis* strain HTCC2156	98%	0.0	95.35%	1438	NR_115159.1
PdG40	30	9	*Brucella anthropi* ATCC 49188	100%	0.0	100.00%	1476	NR_074243.1
PdG41	30	9	*Paenibacillus agaridevorans* strain DSM 1355	100%	0.0	93.76%	1509	NR_025490.1
PdG42	30	9	*Paenibacillus agaridevorans* strain DSM 1355	100%	0.0	93.91%	1509	NR_025490.1
PdG43	40	7	*Brucella anthropi* ATCC 49188	100%	0.0	98.12%	1476	NR_074243.1
PdG44	40	7	*Brucella anthropi* ATCC 49188	98%	0.0	99.53%	1476	NR_074243.1
PdG45	40	7	*Brucella lupini* strain LUP21	97%	0.0	98.86%	1477	NR_042911.1
PdG46	40	9	*Paenibacillus daejeonensis* strain AP-20	100%	0.0	98.11%	1465	NR_104277.1
PdG47	40	9	*Alkalihalobacillus trypoxylicola* strain NBRC 102646	99%	0.0	99.79%	1476	NR_114174.1

PdG48	40	9	*Sutcliffiella horikoshii* strain DSM 8719	100%	3.00e-151	99.32%	1529	NR_119070.1
PdG49	40	9	*Paenibacillus hispanicus* strain AMTAE16	97%	0.0	98.54%	1473	NR_152687.1
PdG50	40	9	*Sutcliffiella horikoshii* strain DSM 8719	100%	0.0	100.00%	1521	NR_040852.1
PdG51	40	9	*Sutcliffiella horikoshii* strain DSM 8719	99%	0.0	99.79%	1521	NR_040852.1
PdG52	40	9	*Paenibacillus hispanicus* strain AMTAE16	100%	0.0	98.11%	1473	NR_152687.1
PdG53	40	9	*Sutcliffiella horikoshii* strain DSM 8719	100%	0.0	96.90%	1529	NR_119070.1
PdG54	40	9	*Paenibacillus hispanicus* strain AMTAE16	99%	0.0	96.72%	1473	NR_152687.1
PdG55	40	9	*Shouchella lehensis* strain MLB2	100%	0.0	99.37%	1525	NR_036940.1
PdG56	40	9	*Shouchella lehensis* strain MLB2	100%	0.0	99.36%	1525	NR_036940.1
PdG57	40	9	*Bacillus horti* strain K13	99%	0.0	98.77%	1512	NR_036860.1
PdG58	40	9	*Paenibacillus hispanicus* strain AMTAE16	100%	0.0	97.26%	1473	NR_152687.1
PdG59	40	9	*Bacillus horti* strain K13	99%	0.0	98.97%	1512	NR_036860.1
PdG60	40	9	*Alkalihalobacillus trypoxylicola* strain NBRC 102646	98%	0.0	99.78%	1476	NR_114174.1
PdG61	40	9	*Alkalihalobacillus trypoxylicola* strain NBRC 102646	98%	0.0	99.57%	1476	NR_114174.1
PdG62	40	9	*Sutcliffiella horikoshii* strain DSM 8719	100%	0.0	99.80%	1521	NR_040852.1
PdG63	40	9	*Alkalihalobacillus trypoxylicola* strain NBRC 102646	98%	0.0	99.79%	1476	NR_114174.1
PdG64	40	9	*Bacillus horti* strain K13	99%	0.0	99.34%	1512	NR_036860.1
PdG65	40	9	*Sutcliffiella cohnii* strain NBRC 15565	95%	0.0	99.58%	1477	NR_113776.1
PdG66	40	9	*Bacillus horti* strain K13	100%	0.0	99.16%	1512	NR_036860.1
PdG67	40	9	*Bacillus horti* strain K13	99%	0.0	98.99%	1512	NR_036860.1
PdG68	40	9	*Bacillus horti* strain K13	98%	0.0	98.75%	1512	NR_036860.1
PdG69	40	9	*Sutcliffiella horikoshii* strain DSM 8719	100%	0.0	100.00%	1521	NR_040852.1
PdG70	40	9	*Bacillus horti* strain K13	98%	0.0	99.18%	1512	NR_036860.1
PdG71	20	7	*Raoultella planticola* ATCC 33531	100%	0.0	99.30%	1401	NR_119214.1
PdG72	20	9	*Aeromonas media* strain ATCC 33907	100%	0.0	100.00%	1460	NR_119041.1

PdG73	20	9	*Aeromonas media* strain ATCC 33907	99%	0.0	100.00%	1460	NR_119041.1
PdG74	30	9	*Raoultella planticola* ATCC 33531 strain DSM 3069	100%	0.0	98.99%	1401	NR_119214.1
PdG75	40	7	*Aeromonas media* strain ATCC 33907	100%	0.0	100.00%	1460	NR_119041.1
PdG76	40	7	*Aeromonas media* strain ATCC 33907	100%	0.0	100.00%	1460	NR_119041.1
PdG77	40	7	*Raoultella planticola* ATCC 33531 strain DSM 3069	99%	0.0	99.35%	1401	NR_119214.1
PdG78	20	9	*Aeromonas media* strain ATCC 33907	100%	0.0	100.00%	1460	NR_119041.1
PdG79	20	9	*Aeromonas media* strain ATCC 33907	100%	0.0	100.00%	1460	NR_119041.1
PdG80	20	9	*Aeromonas media* strain ATCC 33907	100%	0.0	100.00%	1460	NR_119041.1
PdG81	20	9	*Aeromonas media* strain ATCC 33907	100%	0.0	99.79%	1460	NR_119041.1
PdG82	30	9	*Aeromonas media* strain ATCC 33907	99%	0.0	100.00%	1460	NR_119041.1
PdG83	40	9	*Aeromonas media* strain ATCC 33907	100%	0.0	100.00%	1460	NR_119041.1
PdG84	40	9	*Aeromonas media* strain ATCC 33907	100%	0.0	100.00%	1460	NR_119041.1
PdG85	40	9	*Paenibacillus xylanilyticus* strain XIL14	100%	0.0	99.58%	1546	NR_029109.1
PdG86	20	5.5	*Pseudomonas reidholzensis* strain ID3	100%	0.0	100.00%	1531	NR_157777.1
PdG87	20	5.5	*Klebsiella aerogenes* KCTC 2190	100%	0.0	99.23%	1540	NR_102493.2
PdG88	20	5.5	*Raoultella terrigena* strain NBRC 14941	98%	0.0	100.00%	1467	NR_113703.1
PdG89	20	5.5	*Pseudomonas reidholzensis* strain ID3	100%	0.0	100.00%	1531	NR_157777.1
PdG90	20	5.5	*Brucella rhizosphaerae* strain PR17	94%	1.00e-171	95.09%	1387	NR_042600.1
PdG91	20	5.5	*Brucella rhizosphaerae* strain PR17	94%	1.00e-171	95.09%	1387	NR_042600.1
PdG92	20	5.5	*Buttiauxella izardii* strain S3/2-161	100%	0.0	98.73%	1498	NR_025331.1
PdG93	20	7	*Pseudomonas putida* strain ATCC 12633	99%	0.0	98.48%	1492	NR_114479.1
PdG94	20	7	*Klebsiella aerogenes* KCTC 2190	100%	2.00e-143	91.99%	1540	NR_102493.2
PdG95	20	7	*Raoultella terrigena* strain NBRC 14941	98%	0.0	99.13%	1467	NR_113703.1
PdG96			*Pseudomonas putida* strain ATCC 12633	99%	0.0	99.36%	1492	NR_114479.1
PdG97	20	7	*Raoultella terrigena* strain ATCC 33257	100%	0.0	98.93%	1436	NR_114503.1

PdG98	20	9	*Brucella rhizosphaerae* strain PR17	94%	0.0	100.00%	1387	NR_042600.1
PdG99	20	9	*Stenotrophomonas maltophilia* strain IAM 12423	100%	0.0	98.12%	1538	NR_041577.1
PdG100	20	9	*Sphingobacterium cladoniae* strain No.6	100%	0.0	95.77%	1500	NR_108441.1
PdG101	20	9	*Pseudomonas putida* strain ATCC 12633	99%	0.0	99.78%	1492	NR_114479.1
PdG102	20	9	*Stenotrophomonas nitritireducens* strain L2	98%	0.0	99.32%	1513	NR_025305.1
PdG103	30	9	*Sphingobacterium cladoniae* strain No.6	100%	0.0	96.10%	1500	NR_108441.1
PdG104	20	5	*Klebsiella aerogenes* KCTC 2190	100%	0.0	99.57%	1540	NR_102493.2
PdG105	20	5	*Klebsiella aerogenes* KCTC 2190	100%	0.0	99.16%	1540	NR_102493.2
PdG106	20	5	*Klebsiella aerogenes* KCTC 2190	100%	0.0	98.98%	1540	NR_102493.2
PdG107	20	5	*Raoultella terrigena* strain NBRC 14941	100%	0.0	99.74%	1467	NR_113703.1
PdG108	20	5	*Klebsiella aerogenes* KCTC 2190	100%	0.0	98.83%	1540	NR_102493.2
PdG109	20	5	*Klebsiella aerogenes* KCTC 2190	100%	0.0	99.58%	1540	NR_102493.2
PdG110	20	7	*Klebsiella aerogenes* KCTC 2190	100%	0.0	99.73%	1540	NR_102493.2
PdG111	20	7	*Raoultella terrigena* strain NBRC 14941	100%	0.0	98.24%	1467	NR_113703.1
PdG112	20	7	*Raoultella terrigena* strain NBRC 14941	100%	0.0	98.24%	1467	NR_113703.1
PdG113	20	7	*Klebsiella aerogenes* KCTC 2190	100%	0.0	99.57%	1540	NR_102493.2
PdG114	20	7	*Raoultella terrigena* strain NBRC 14941	100%	0.0	100.00%	1467	NR_113703.1
PdG115	20	9	*Klebsiella aerogenes* KCTC 2190	100%	0.0	99.49%	1540	NR_102493.2
PdG116	20	9	*Raoultella terrigena* strain NBRC 14941	100%	0.0	99.74%	1467	NR_113703.1
PdG117	20	5	*Klebsiella aerogenes* KCTC 2190	100%	0.0	99.24%	1540	NR_102493.2
PdG118	20	5	*Raoultella terrigena* strain ATCC 33257	100%	0.0	99.33%	1436	NR_114503.1
PdG119	20	5	*Klebsiella aerogenes* KCTC 2190	100%	0.0	99.57%	1540	NR_102493.2
PdG120	30	5	*Raoultella terrigena* strain NBRC 14941	99%	0.0	99.30%	1467	NR_113703.1
PdG121	30	5	*Brucella rhizosphaerae* strain PR17	94%	0.0	100.00%	1387	NR_042600.1
PdG122	30	7	*Klebsiella aerogenes* KCTC 2190	100%	0.0	98.83%	1540	NR_102493.2

PdG123	30	7	*Klebsiella aerogenes* KCTC 2190	99%	0.0	99.35%	1540	NR_102493.2
PdG124	30	7	*Klebsiella aerogenes* KCTC 2190	100%	0.0	99.37%	1540	NR_102493.2
PdG125	30	9	R*aoultella terrigena* strain ATCC 33257	100%	0.0	98.93%	1436	NR_114503.1
PdG126	30	9	*Brucella anthropi* ATCC 49188	100%	0	99.75%	1476	NR_074243.1
PdG127	30	9	*Raoultella terrigena* strain NBRC 14941	98%	0.0	99.77%	1467	NR_113703.1
PdG128	40	5	*Raoultella terrigena* strain NBRC 14941	100%	0.0	98.86%	1467	NR_113703.1

(1) Per. Ident. – percentage of identity
(2) Acc. Len. – accession length
